# Cross-Linked Poly(methyl methacrylate) Nanocomposites’ Synthesis, Characterization, and Antibacterial Effects

**DOI:** 10.3390/polym17030269

**Published:** 2025-01-21

**Authors:** Nazeeha S. Alkayal, Mashail A. Al Ghamdi

**Affiliations:** 1 Chemistry Department, Faculty of Science, King Abdulaziz University, P.O. Box 80203, Jeddah 21589, Saudi Arabia; 2 Biological Department, Faculty of Science, King Abdulaziz University, P.O. Box 80200, Jeddah 21589, Saudi Arabia

**Keywords:** cross-linked PMMA, melamine, CuO nanoparticle, activated carbon, antibacterial, *E. coli*, *S. aureus*

## Abstract

Polymer networks were synthesized using the condensation method between PMMA and melamine as cross-linkers. CuO nanoparticles (NPs) and activated carbon (AC) were used as a filler. The final products PMMA/Mel, PMMA/Mel-CuO, and PMMA/Mel-AC were tested for antibacterial activities against *E.coli* and *S. aureus.* The chemical structure and composition, thermal properties, and surface morphology of the new PMMA/Mel-based nanocomposites were investigated by various techniques. The XRD and EDX results showed the successful incorporation of CuO NPs and AC into the polymer matrix. Also, the thermal stability of the PMMA/Mel polymer was significantly enhanced after adding CuO nanoparticles. This finding showed that the PMMA/Mel-CuO and PMMA/Mel-AC nanocomposites have greater activity against both bacteria than PMMA/Mel. The PMMA/Mel-CuO and PMMA/Mel-AC polymers showed high activity against *S. aureus* bacteria, with inhibition zones of 22.6 mm and 11.3 mm, respectively. This confirms that small-sized nanoparticles have an effective role in killing bacterial cells.

## 1. Introduction

Microorganisms have an important role in regulating all living things, including humans, via biogeochemical processes. Infection occurs when a microbe escapes or impairs the host’s defense mechanisms; such germs are termed pathogenic bacteria [1,2]. Bacterial resistance to antibacterial drugs occurs as a result of genetic changes caused by the misuse and overuse of antibiotics [3]. As a result, there is a compelling need to create novel critical antibacterial agents to combat antibiotic resistance.

PMMA (poly methyl methacrylate) has been utilized as an antibacterial substance in the medical industry over the last three-quarters of a century. Nonetheless, its antibacterial properties remain insufficient; when carrying out anatomical restoration and tissue implanting for tissue regeneration, biomaterial-derived infections can occur once the materials are attached to the body [4,5]. The development of biomaterials using nanotechnology is seen as a strong option that has lately been used to overcome this barrier.

To treat bacterial infections, different nanoparticles with antibacterial properties have emerged as a new class of biological chemicals, which have a strong influence on bacterial proliferation [6]. As part of this trend, PMMA was combined with metals, metal oxides, and carbon-based nanoparticles to create nanocomposites with enhanced antibacterial properties [7]. In this regard, PMMA was treated with cross-linking agents to generate a porous skeleton, which was then filled with nanoparticles. Antibacterial drugs hinder cell wall and protein production, DNA damage, and bacterial metabolism, all of which end in bacterial death [8].

According to the literature, copper oxide (CuO) has several functions, including antibacterial capabilities [9,10,11,12,13,14,15,16,17,18]. Copper oxide nanostructures have been shown to have higher antibacterial activity than other metal oxides [19,20]. Furthermore, they are less costly than other metals such as Ag, may be readily disseminated in polymeric materials, and are used as implant substances [21]. In the same vein, low-cost activated carbon has outstanding antibacterial action and can be simply produced from biowaste-derived compounds [22,23,24,25]. It has a greater surface area, homogenous particle size, biocompatibility, and environmental friendliness, all of which contribute to its excellent bacterial adsorption efficiency [26].

Research studies have revealed the antibacterial effects of several nanoparticles. For example, Giti et al. discovered that adding CuO nanoparticles at concentrations of 2.5% and 7.5% to PMMA resin improves its antibacterial efficacy against *S. salivarius*, *S. sanguis*, and *C. dubliniensis* bacterial strains [27]. Sathya et al. created a PMMA nanocomposite with 0.1% *w*/*v* copper oxide NPs (PMMA-CuO), which showed antibacterial efficacy against *E. coli*. Meanwhile, a cetyl trimethyl ammonium bromide-capped CuO (PMMA-CuO (CTAB)) nanocomposite (0.1% *w*/*v*) was extremely effective against *S. aureus* [28]. Glazkova et al. created a 5 wt.% CuFe_2_O_4_/Cu_2_O/CuO/PMMA nanocomposite, which showed 100% and 99.94% antibacterial effectiveness against *E. coli* and MRSA bacterial strains, respectively [29]. Varghese et al. investigated the synthesis of activated carbon from kitchen soot as a biowaste [30]. In their work, the antibacterial activity of AC nanoparticles was tested against both Gram-negative and Gram-positive bacterial strains with inhibition zones ranging from 19 to 29 mm. In another study, AC was produced from the Passiflora foetida plant and evaluated against twelve different bacterial strains using an agar-well diffusion technique. The data showed that AC has effective antibacterial action against practically all microorganisms [31].

In the present research, we prepared PMMA/Mel-CuO and PMMA/Mel-AC nanocomposites using a polycondensation reaction, and their structural and morphological characteristics were investigated by TGA, FT-IR, SEM, XRD, and elemental analysis to confirm the chemical distribution. The biological properties of the two nanocomposites were studied and compared with the cross-linked PMMA/Mel without NPs. All of the previous research findings were considered in our investigation, exploration, and implementation of the composite when exposed to *E. coli* as a Gram-negative and resistant bacteria. Our findings suggest that this is the optimum polymer composite to apply against *E. coli* gastrointestinal tract infections.

## 2. Experimental Section

### 2.1. Materials

Alfa Aesar (Haverhill, MA, USA) provided poly (methyl methacrylate) (PMMA, M_wt_ = 300 k), while BDH laboratory reagents supplied dimethyl sulfoxide (DMSO 99%) and melamine (97.5%). The Nano Center provided activated carbon and copper oxide nanoparticles (CuO < 100 nm). All compounds were utilized without additional purification.

### 2.2. Instrumentation

Thermogravimetric analysis (TGA) was carried out using a TGA4724 at a heating rate of 10 °C/min between 25 and 500 °C in a N_2_ environment. The Nicolet Magna 6700 FT spectrometer (Thermo Fisher Scientific, Waltham, MA, USA) was used to examine the Fourier transform infrared spectra (FTIR) in the 400–4000 cm^−1^ range. X-ray diffraction (XRD) patterns were analyzed using a Bruker D8 Advance and Cu Kα radiation (wavelength 1.5418 Å) at 40 kV and 40 Ma (Bruker, Billerica, MA, USA). The patterns were gathered between 2θ of 10° and 60°, with a scan speed of 1.5°/min. Imaging was performed using an FEI TENEO VS microscope with an EDAX detector (Thermo Fisher Scientific, Waltham, MA, USA). To prevent sample charging during imaging, the polymer sample was attached to an aluminum stub with sticky carbon tape and sputter coated with 3 nm iridium.

### 2.3. Synthesis of PMMA/Mel, PMMA/Mel-CuO, and PMMA/Mel-AC Nanocomposites

PMMA/Mel was created in accordance with the prior work [32], whereby 1 g of PMMA and 20% w/w melamine were added to 30 mL DMSO and heated to 175 °C for 72 h. After cooling, the product was filtered and washed with methanol, THF, and dichloromethane, yielding 85%.

PMMA/Mel-CuO and PMMA/Mel-AC nanocomposites were created utilizing in situ polymerization. Here, 1 g of PMMA was dissolved in 30 mL DMSO, and 0.1 g of CuO nanoparticles was stirred into the PMMA solution before being ultrasonically agitated for 10 min. Melamine was then added as a cross-linker, and the mixture was refluxed for 72 h at 175 °C with constant shaking. Following cooling, the product was filtered and washed with methanol, THF, and di-chloromethane, yielding 87%. The assay of each drug/antibiotic’s loaded content was performed using UV-spectroscopy on the suitable Lambda max (Shimadzu, Kyoto, Japan).

### 2.4. The Antibacterial Activities of PMMA/Mel-CuO and PMMA/Mel-AC Nanocomposites and PMMA/Mel

#### 2.4.1. Preparation of the Inoculums

Two fresh strains of bacteria were used in the experiments; one was *Staphylococcus aureus* as a Gram-positive bacterium, and the other was *Escherichia coli* as a Gram-negative bacterium. These were cultured on Muller–Hinton agar plates, and inoculums were prepared from a 24 h old culture and then incubated overnight at 37 °C. The inoculums from both strains were inoculated and suspended in 2 different 50 mL sterile Muller–Hinton broths by sterile cotton swapping. The absorbance (at 540 nm) was determined and measured for the 2 cultures after the incubation period at 37 °C. Then, the OD was adjusted to reach 1.5 × 10^8^ CFU/mL of bacterial cells [33].

#### 2.4.2. Determination of the Antibacterial Activity of (PMMA) Polymers Using the Agar-Well Diffusion Method

Evaluation of the antibacterial activity of three polymer solutions was performed with the agar-well diffusion method [33]. In a sterile water McFarland tube, 100 μL of each previous fresh bacterial culture was suspended to reach 1.5 × 10^8^ CFU/mL as the standard value of the bacterial count. The bacterial suspension was spread over an MHA plate using a sterilized swab. Three holes with a diameter of 6 mm were punched by a sterile cork-borer in every bacterial culture plate. Then, 0.1 g of each polymer was suspended in 0.5 mL of DMSO and vortexed for 5 min. About 50 μL of each polymer was added to each well. After 18–24 h of incubation at 37 °C, the halo (clear) zones of inhibition of bacterial growth were then measured in millimeters. The above technique was performed in triplicate for the suggested replications. Lastly, we inoculated a plate of *E. coli* and *S. aureus* and applied different antibiotics to determine their resistance to the bacteria in MHA media when incubated for 24 h at 37 °C.

#### 2.4.3. Antibiotics

CPM: Cefepime, GM: Gentamycin, C: Chloramphenicol, OX: Oxacillin, VA: Vancomycin, FC: Fusidic Acid, TS: Trimethoprim/Sulfamethoxazole, CD: Clindamycin, KF: Cephalothin, AP: Amoxicillin/Clavulanic Acid, E: Erythromycin, PG: Penicillin.

#### 2.4.4. Antibiotic Sensitivity Assay for Bacteria

*E. coli* pathogenic bacteria was tested against twelve antibiotics to determine their susceptibility. The bacteria were cultured overnight in nutritional broth for activation, determined by measuring the optical density (OD) at 600 nm with a UV-visible spectrophotometer, and, subsequently, standardized to 0.1 McFarland standards (3107 CFU/mL). Later, the bacterial suspension was dispersed on MHA. The agar was then covered with antibiotic discs and incubated at 37 °C for 24 h. Later, the sizes of the zones of inhibition were measured and reported [34].

#### 2.4.5. Determination of the Antibacterial Activity of (PMMA) Polymers Using the Colony Count Method

The antibacterial activity levels of the PMMA/Mel nanocomposite polymers were studied by the viable cell colony count method (CFU) against *E. coli*. Firstly, all sample of polymers were immersed in 4 mL nutrient broth containing ∼ 10^6^–10^7^ colony-forming units per mL (CFU/mL). Then, the solution was shaken at 37 °C. A flask containing bacteria and no sample was used as the control. After 2 h of incubation, 0.2 mL of bacterial culture was removed from the flask, and serial dilutions were repeated with each initial sample. Then, 50 μL diluent of the sample was spread on MHA plates and incubated at 37 °C for 24 h. The number of viable microorganism colonies was counted manually using a pen and a click-counter and multiplied by the dilution factor. The percentage of inhibition of each film was calculated with the following equation (ASTM E2149-20) [35]:

Mortality(%) = B − A/ B × 100

where B and A are the mean numbers of bacteria in the control samples (CFU/sample) and treated samples after 24 h of incubation (CFU/sample), respectively. The above technique was performed in triplicate for the suggested replications [36].

## 3. Results and Discussion

### 3.1. Structural Investigation

**Figure 1:**
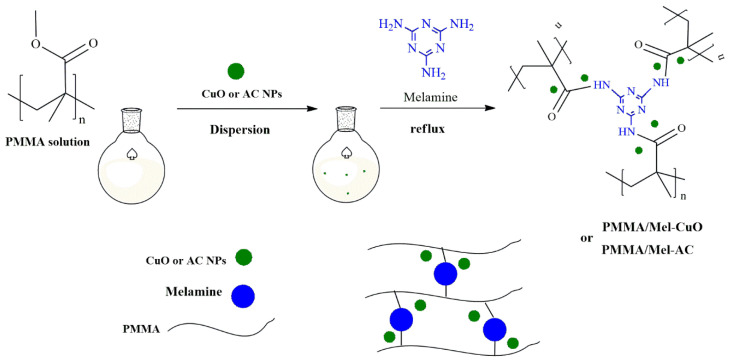
Preparation of PMMA/Mel-CuO and PMMA/Mel-AC nanocomposites.

As described in our previous research article [32], the linear PMMA was chemically reacted using the polycondensation reaction with melamine as a cross-linking agent to form a cross-linked PMMA/Mel polymer network. Then, the prepared PMMA/Mel was incorporated by adding CuO and activated carbon nanoparticles to produce PMMA/Mel-CuO and PMMA/Mel-AC nanocomposites. Figure 1 gives a schematic representation of the methods for preparing the PMMA/Mel-CuO and PMMA/Mel-AC nanocomposites.

**Figure 2:**
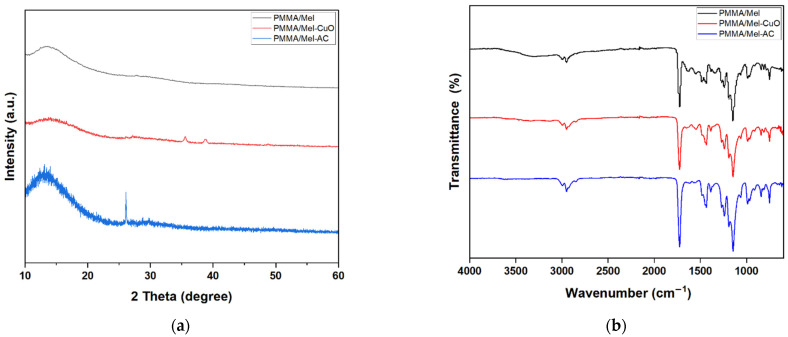
(**a**) FTIR spectra and (**b**) XRD patterns of PMMA/Mel, PMMA/Mel-CuO, and PMMA/Mel-AC.

The FT-IR spectrum was investigated in the range of 600–4000 cm^−1^ and is presented in Figure 2a. The formation of PMMA/Mel cross-linked polymer was confirmed by the appearance of a band at 1700 cm^−1^ of amide carbonyl functional groups. Also, other bands at around 3000 cm^−1^ and 1100 cm^−1^ were noted, which are related to the N-H bond of the stretching and bending vibrations of the secondary amide, as illustrated in our previous work. This indicates successful condensation between melamine monomers (the cross-linker) and the linear PMMA. After the addition of nanoparticles, i.e., CuO and AC, no obvious change was observed in the FT-IR spectra, suggesting that there are no covalent bonds formed between PMMA/Mel and NPs [29].

The X-ray diffraction patterns of PMMA/Mel were analyzed before and after the addition of CuO and AC nanoparticles, and the findings are shown in Figure 2b. The XRD diffractograms of the samples were studied in the 2-theta range between 10° and 60°. For all samples, a broad peak was observed at around 13° related to the PMMA/Mel matrix. The absence of any sharp peaks in the PMMA/Mel pattern showed that the polymer matrix is amorphous. New sharp peaks appeared in the PMMA/Mel-CuO curve at 35.5°, 38.7°, and 48.8°, which match the XRD pattern of standard copper oxide (JCPDS 48–1548), confirming the incorporation of CuO NPs into polymer segments to form a semicrystalline nanocomposite [37]. The XRD pattern of PMMA/Mel-AC showed an intense and sharp peak at 26°, which could be assigned to a particular crystal phase of the activated carbon nanoparticles, indicating the successful attachment of AC NPs with the polymer.

### 3.2. Thermal Study

**Figure 3:**
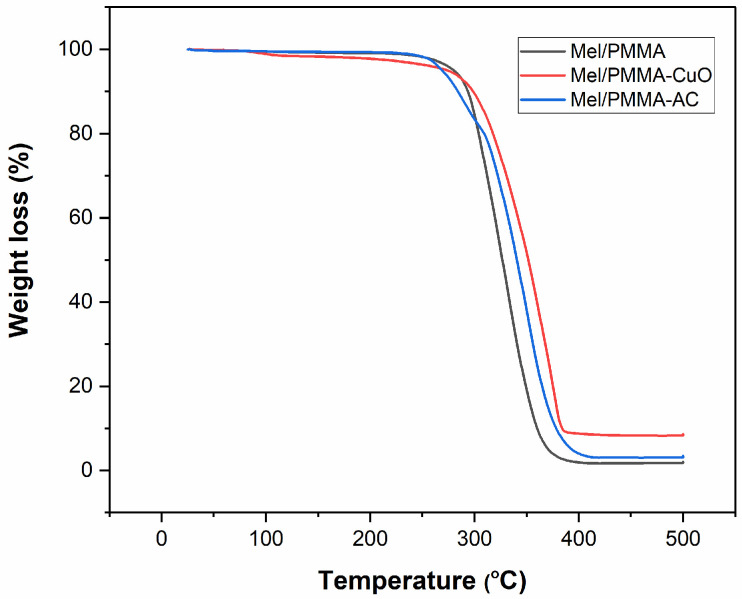
TGA micrograph of cross-linked PMMA/Mel, PMMA/Mel-CuO, and PMMA/Mel-AC.

As presented in Figure 3, the thermal stability levels of the cross-linked PMMA/Mel, PMMA/Mel-CuO, and PMMA/Mel-AC polymers were studied using the TGA technique. The TGA curve for PMMA/Mel showed that degradation takes place primarily in a single step at temperatures ranging from 270 °C to 350 °C. The results demonstrated a significant improvement in the thermal stability of the PMMA/Mel-CuO and PMMA/Mel-AC nanocomposites compared to PMMA/Mel, with degradation occurring at 300 °C. The addition of metal oxides to the polymer network has a positive impact in increasing thermal stability [38]. Table 1 illustrates the decomposition percentages (T _50_, T_30_, and T_10_), which define the temperatures for mass losses of 50%, 30%, and 10%, respectively. T_50_, T_30_, and T_10_ of PMMA/Mel-CuO were higher than those of the cross-linked PMMA/Mel and PMMA/Mel-AC.

### 3.3. Morphological Investigation

**Figure 4:**
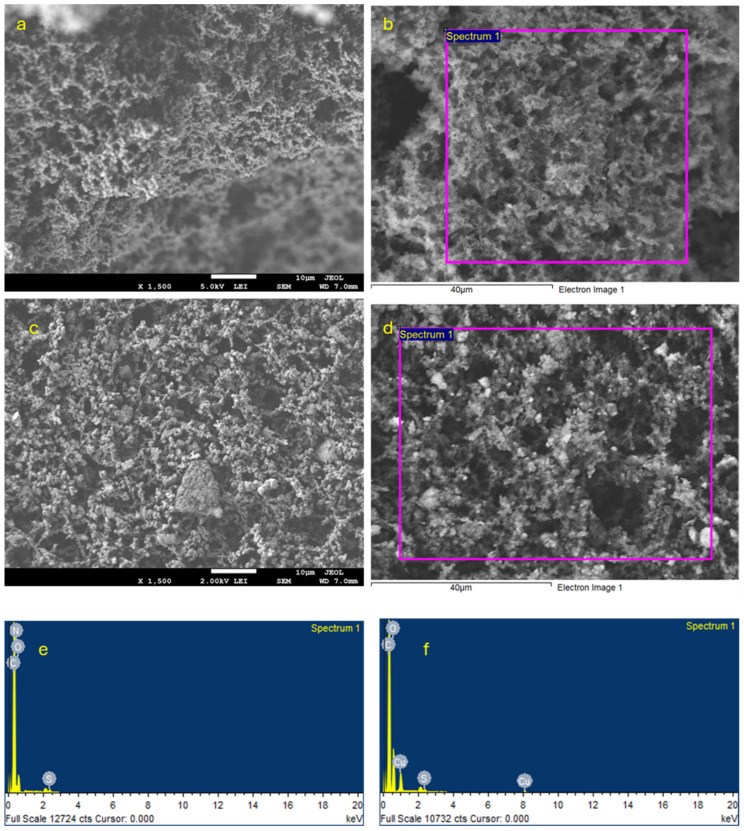
SEM micrographs for (**a**,**b**) PMMA/Mel-AC and (**c**,**d**) PMMA/Mel-CuO, and EDX elemental mapping of (**e**) PMMA/Mel-AC and (**f**) PMMA/Mel-CuO.

The surface morphologies of PMMA/Mel, PMMA/Mel-CuO, and PMMA/Mel-AC were investigated using the SEM method, as displayed in Figure 4. In the micrometer range, the cross-linked PMMA/Mel polymer was revealed to have a rough, porous surface containing small, disorganized particles (Figure 4a,b). After surface modifications with NPs, the surface structure of PMMA/Mel was preserved (Figure 4c–f).

EDX analysis was conducted for elemental mapping of the nanocomposites, as shown in Figure 4 and Table 2. The elemental contents of the PMMA/Mel-CuO spectrum, given in Figure 4f, showed peaks for the O, C, and Cu elements, and the PMMA/Mel-AC spectrum (Figure 4e) exhibited peaks for the N, O, and C elements, confirming the formation of the nanocomposite.

### 3.4. Antibacterial Activity of Polymers Determined with the Agar-Well Diffusion Method

The antibacterial activity for the three polymers was measured with agar-well diffusion methods. In Table 3, the figures demonstrate that PMMA/Mel-CuO NP polymers had great antibacterial activity, with a clear zone from 8.3 ± 0.09 to 22.6 ± 0.1 mm against the tested bacteria. Meanwhile, the cross-linked PMMA/Mel, with a clear zone from 9.6.0 ± 0.02 to 8.3 ± 0.09 mm, showed the lowest antibacterial effect against the tested bacteria. Similarly, PMMA/Mel-AC produced the same result, with a clear zone measured from 8.6 ± 0.1 to 11.3 ± 0.1 mm. In this study, all tested polymers showed efficient activity against both *E. coli* and *S. aureus* strains. The PMMA/Mel-CuO polymer showed high activity against *S. aureus* (22.6 mm), followed by PMMA/Mel-AC with a 11.3 mm zone.

**Figure 5:**
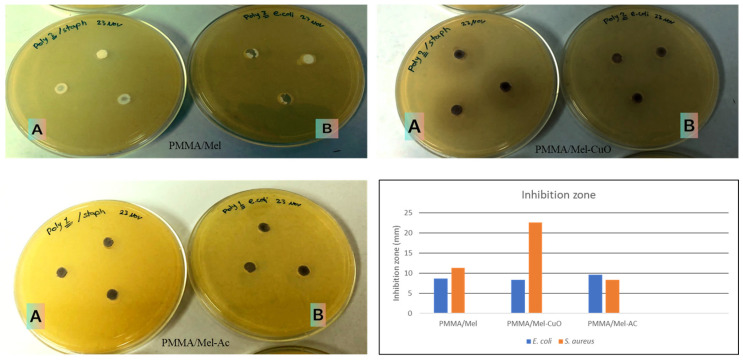
The antibacterial activity of cross-linked PMMA/Mel, PMMA/Mel-CuO, and PMMA/Mel-AC against (**A**) *S. aureus* and (**B**) *E. coli*.

In the present study, through a suitable diffusion method, PMMA polymers and their activity were tested as antibacterial agents against *S. aureus* as a Gram-positive bacterium and *E.coli* as a Gram-negative bacterium. In Figure 5, it can be seen that PMMA/Mel showed antimicrobial efficient activity against the two different bacterial strains, which was promoted by increasing the integration with NPs. The PMMA/Mel polymer, with the incorporation of CuO and activated carbon NPs, had greater activity against the bacteria than PMMA/Mel without the integration with NPs, as shown in Figure 5. This enhancement in the antibacterial activity arises from the very small size of CuO NPs, which can enter the cell wall of the bacterium and then kill it via denaturation of the cell permeability, or from the contact of CuO with many enzymes.

In alignment with previous studies [39,40], microbial adhesion on PMMA-included biomaterial was reduced when PMMA was incorporated with activated carbon, and PMMA nanofibers showed good antibacterial activity against some pathogenic bacterial strains. In regard to the stability of polymers and their difficult solubility in media, PMMA/Mel incorporation with NPs increased the clear zone of bacterial growth. In another study, PMMA incorporated with metronidazole and chlorhexidine showed promising antibacterial activity against *Enterococcus faecalis* but not against *Porphyromonas gingivalis* [41].

### 3.5. Antibiotic Sensitivity Assay of Pathogenic E. coli Strain

**Figure 6:**
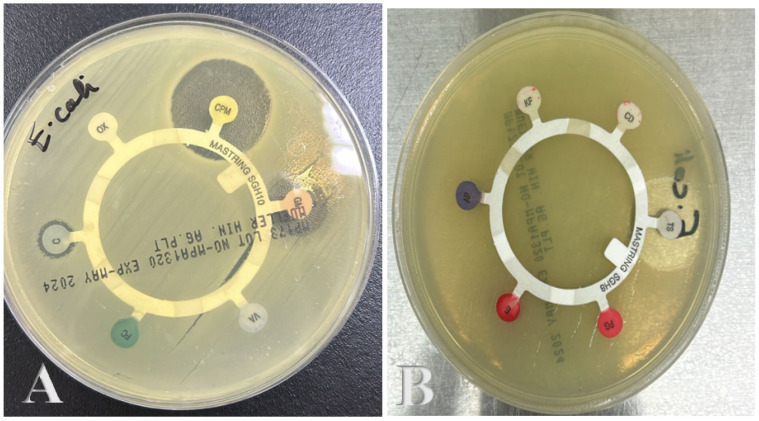
(**A**,**B**) Antibiotic resistance profiles of *E. coli* pathogenic bacteria. Antibiotic compounds used; CPM: Cefepime, GM: Gentamycin, C: Chloramphenicol, OX: Oxacillin, VA: Vancomycin, FC: Fusidic Acid, TS: Trimethoprim/Sulfamethoxazole, CD: Clindamycin, KF: Cephalothin, AP: Amoxicillin/Clavulanic Acid, E: Erythromycin, PG: Penicillin.

In the present study, when we applied pathogenic *E. coli*, a positive result was observed as an inhibitory zone surrounding the bacterial colonies. The disc diffusion susceptibility test was carried out to determine the sensitivity or resistance of pathogenic microorganisms to various antimicrobial agents. On Mueller–Hinton agar, the pathogenic bacteria were cultivated in the presence of antimicrobial-impregnated filter paper discs. The presence or absence of bacterial growth around the discs is regarded as an indirect sign of the compound’s ability to inhibit the organism. The results of the antibiotic sensitivity assay revealed a significant disparity between antibiotic resistance and sensitivity among the tested bacterial strains. Several antibiotics demonstrated resistance, including OX, VA, FC, CD, KF, AP, E, and P. In contrast, certain antibiotics showed measurable sensitivity, with TS exhibiting the highest sensitivity at 23 mm, followed by CPM at 20 mm, GM at 15 mm, and C at 4 mm. These findings indicate that while some antibiotics are ineffective against these bacterial strains, others may still hold therapeutic potential. Table 4 and Figure 6A,B illustrate the antibiotic resistance characteristics of the pathogenic bacterial strain. The antibiotics TS and CPM produced inhibitory regions, indicating their effectiveness in halting bacterial growth. However, it is also evident that some compounds exhibited resistance when tested against *E. coli*. This resistance highlights the adaptive mechanisms of *E. coli*, necessitating further investigation into the factors contributing to such resistance and the potential development of alternative therapeutic strategies [42].

### 3.6. Antibacterial Activity of Polymers Determined with the Colony Count Method

**Figure 7:**
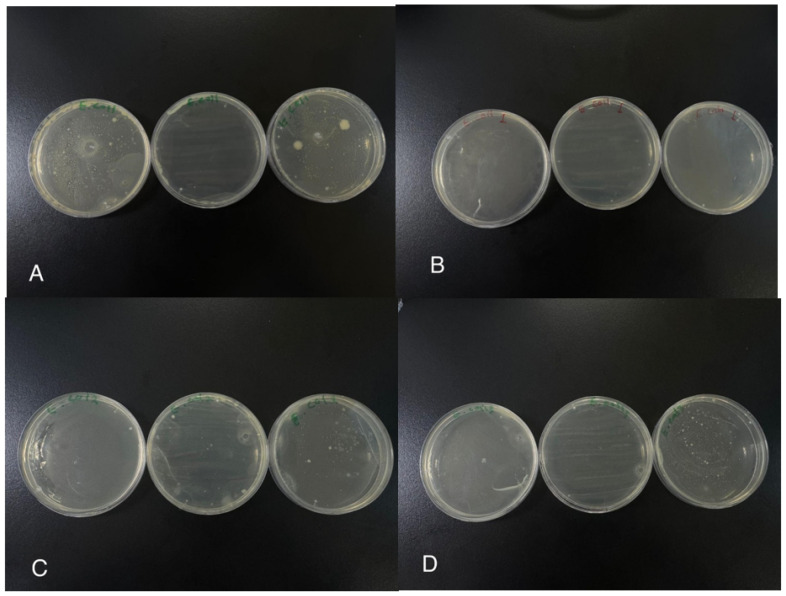
(**A**–**D**): The antibacterial activity of cross-linked (**B**) PMMA/Mel, (**C**) PMMA/Mel-CuO, and (**D**) PMMA/Mel-AC against *E. coli* according to the colony-forming units compared to (**A**) *E.coli* as a control.

The antibacterial activities of the three polymers were also tested by the CFU counting method to confirm the results obtained by the well diffusion method. The results showed that polymers impacted the number of *E. coli* colonies, leading to different numbers compared to the control, as can be seen in Table 5 and Figure 7. The results showed decreases in the average number of colonies compared to the control dish’s 27.33 CFU. The average colony number in the sample treated with PMMA and Mel was approximately 1.67 CFU, with a high killing mortality of 90%. Meanwhile, the mean in the sample treated with PMMA, Mel, and CuO was approximately 17.33 CFU, with 37% mortality. In the sample treated with PMMA, Mel, and Ac, it was approximately 8.33 CFU, with 70% mortality.

A culture medium without any sample was used as a control. The antibacterial activity of polymers against Gram-negative bacteria may arise from interaction between active groups of polymers and anionic lipopolysaccharides of the Gram-negative cells, causing cell wall destruction, leakage of intracellular components, and inhibited transport of nutrients into the cells, finally leading to cell death [29]. The addition of polymethyl methacrylate (PMMA) to nanoparticles (NPs) reduces their efficiency in killing bacteria. This is because PMMA tends to encapsulate nanoparticles, preventing direct contact with bacterial cells and diminishing antibacterial mechanisms like reactive oxygen species (ROS) generation and ion release. Additionally, PMMA can cause nanoparticles to agglomerate [29], reducing their effective surface area for interaction with bacteria. The polymer matrix may also weaken the nanoparticles’ effects, making it harder for them to penetrate bacterial membranes or disrupt vital biochemical processes. In another study, PMMA incorporation with vancomycin and gentamicin reduced the number of colony-forming units per mL (*p* < 0.05). Gentamicin-loaded PMMA could inhibits the growth of sessile cells (*p* < 0.05), and the group with vancomycin 4 g + gentamicin 500 mg presented the best results [43].

## 4. Conclusions

In this work, the PMMA/Mel polymer network was incorporated with CuO and AC NPs through polycondensation reactions, and the products were employed as antibacterial agents against *S. aureus* as a Gram-positive bacterium and *E. coli* as a Gram-negative bacterium. The chemical structure and composition, thermal properties, and surface morphology of the new PMMA/Mel-based nanocomposites were investigated by various techniques. The XRD and EDX results showed the successful incorporation of CuO and AC NPs into the polymer matrix. Also, the thermal stability of the PMMA/Mel polymer was significantly enhanced after adding CuO nanoparticles. The finding showed that the PMMA/Mel-CuO and PMMA/Mel-AC nanocomposites had greater activity against both bacteria than PMMA/Mel. PMMA/Mel-CuO and PMMA/Mel-AC polymers showed high activity against *S. aureus* bacteria, with inhibition zones of 22.6 mm and 11.3 mm, respectively. This confirms that small-sized nanoparticles have an effective role in killing bacterial cells.

## Figures and Tables

**Table 1:** Thermal properties of cross-linked PMMA/Mel, PMMA/Mel-CuO, and PMMA/Mel-AC.

Sample	T_10_	T_30_	T_50_
PMMA/Mel	305	311	326
PMMA/Mel-CuO	315	340	360
PMMA/Mel-AC	298	330	345

**Table 2:** The elemental composition of PMMA/Mel-CuO and PMMA/Mel-AC samples.

Sample	PMMA/Mel-CuO				PMMA/Mel-AC			
Element	C K	O K	S K	Cu L	C K	N K	O K	S K
Weight%	63.69	28.33	0.51	7.47	59.34	20.07	19.99	0.60
Atomic%	73.58	24.57	0.22	1.63	64.66	18.75	16.35	0.24

**Table 3:** Antimicrobial activity (mm) of polymers determined using the agar-well diffusion method.

Polymers	Inhibition Zone (mm)	
	*E. coli*	*S. aureus*
PMMA/Mel	9.6	8.3
PMMA/Mel-CuO	8.3	22.6
PMMA/Mel-AC	8.6	11.3

Data are expressed as the mean ± SD (*n* = 3).

**Table 4:** Susceptibility of the *E. coli* strain used in the present study to different antibiotics.

Antibiotic	Resistance or Inhibitory Zone (mm) Produced by Antibiotics
CPM	20
GM	15
C	4
OX	R *
VA	
FC	
TS	23
CD	R
KF	
AP	
E	
PG	R

* R: Resistance. Antibiotic compounds used; CPM: Cefepime, GM: Gentamycin, C: Chloramphenicol, OX: Oxacillin, VA: Vancomycin, FC: Fusidic Acid, TS: Trimethoprim/Sulfamethoxazole, CD: Clindamycin, KF: Cephalothin, AP: Amoxicillin/Clavulanic Acid, E: Erythromycin, PG: Penicillin.

**Table 5:** Antimicrobial activity of polymers on *E. coli* determined using the colony count method.

	CFU	Mortality%
Control	27.33 ± 16	-
PMMA/Mel	1.6 ± 1.5	94%
PMMA/mel/CuO	17.3 ± 18	37%
PMMA/Mel/Ac	8.3 ± 10.11	70%

Control: *E. coli.*

## Data Availability

Data are contained within the article.
